# The intracellular bacterium *Anaplasma phagocytophilum* selectively manipulates the levels of vertebrate host proteins in the tick vector *Ixodes scapularis*

**DOI:** 10.1186/s13071-016-1747-3

**Published:** 2016-08-25

**Authors:** Margarita Villar, Vladimir López, Nieves Ayllón, Alejandro Cabezas-Cruz, Juan A. López, Jesús Vázquez, Pilar Alberdi, José de la Fuente

**Affiliations:** 1 SaBio. Instituto de Investigación en Recursos Cinegéticos IREC-CSIC-UCLM-JCCM, Ronda de Toledo s/n, 13005, Ciudad Real, Spain; 2 University Lille, CNRS, Inserm, CHU Lille, Institut Pasteur de Lille, U1019 - UMR 8204 - CIIL - Centre d’Infection et d’Immunité de Lille, F-59000 Lille, France; 3 Centro Nacional de Investigaciones Cardiovasculares (CNIC), Madrid, Spain; 4 Department of Veterinary Pathobiology, Center for Veterinary Health Sciences, Oklahoma State University, Stillwater, OK 74078 USA

**Keywords:** *Anaplasma*, Tick, *Ixodes*, Proteomics, Hemoglobin, Immunology

## Abstract

**Background:**

The intracellular bacteria *Anaplasma phagocytophilum* are emerging zoonotic pathogens affecting human and animal health, and a good model for the study of tick-host-pathogen interactions. This tick-borne pathogen is transmitted by *Ixodes scapularis* in the United States where it causes human granulocytic anaplasmosis. Tick midguts and salivary glands play a major role during tick feeding and development, and in pathogen acquisition, multiplication and transmission. Vertebrate host proteins are found in tick midguts after feeding and have been described in the salivary glands of fed and unfed ticks, suggesting a role for these proteins during tick feeding and development. Furthermore, recent results suggested the hypothesis that pathogen infection affects tick metabolic processes to modify host protein digestion and persistence in the tick with possible implications for tick physiology and pathogen life-cycle.

**Methods:**

To address this hypothesis, herein we used *I. scapularis* female ticks fed on uninfected and *A. phagocytophilum*-infected sheep to characterize host protein content in midguts and salivary glands by proteomic analysis of tick tissues.

**Results:**

The results evidenced a clear difference in the host protein content between tick midguts and salivary glands in response to infection suggesting that *A. phagocytophilum* selectively manipulates the levels of vertebrate host proteins in ticks in a tissue-specific manner to facilitate pathogen infection, multiplication and transmission while preserving tick feeding and development. The mechanisms by which *A. phagocytophilum* manipulates the levels of vertebrate host proteins are not known, but the results obtained here suggested that it might include the modification of proteolytic pathways.

**Conclusions:**

The results of this study provided evidence to support that *A. phagocytophilum* affect tick proteolytic pathways to selectively manipulate the levels of vertebrate host proteins in a tissue-specific manner to increase tick vector capacity. Investigating the biological relevance of host proteins in tick biology and pathogen infection and the mechanisms used by *A. phagocytophilum* to manipulate host protein content is essential to advance our knowledge of tick-host-pathogen molecular interactions. These results have implications for the identification of new targets for the development of vaccines for the control of tick-borne diseases.

**Electronic supplementary material:**

The online version of this article (doi:10.1186/s13071-016-1747-3) contains supplementary material, which is available to authorized users.

## Background

Infectious diseases transmitted by arthropod vectors such as ticks constitute a growing burden for human and animal health worldwide [1–4]. *Anaplasma phagocytophilum* (Rickettsiales: Anaplasmataceae) are tick-borne intracellular bacteria that infect vertebrate host granulocytes causing human, canine and equine granulocytic anaplasmosis and tick-borne fever of ruminants [5–8]. Human granulocytic anaplasmosis (HGA) is the second most common tick-borne disease in the United States, and tick-borne fever is an established and economically important disease of sheep in Europe [6, 9]. This emerging zoonotic pathogen is transmitted by *Ixodes* ticks of which the major vector species are *I. scapularis* in the United States and *I. ricinus* in Europe [2]. In ticks, *A. phagocytophilum* infects and multiply in different tissues including midguts [10] and salivary glands [11].

*Anaplasma phagocytophilum* is a good model for the study of tick-host-pathogen interactions because recent results have shown that infection affects gene expression and protein levels in vertebrate hosts and ticks [8, 12]. Recently, we proposed that the evolution of *I. scapularis*-host-*A. phagocytophilum* molecular interactions involving genetic traits of all parts resulted in conflict and cooperation between them, with mutual beneficial effects for ticks, hosts and pathogens [13]. Furthermore, *A. phagocytophilum* evolved common strategies for infection of vertebrate host and tick cells that include but are not limited to remodeling of the cytoskeleton, inhibition of cell apoptosis, manipulation of the immune response and control of host cell epigenetics [14].

Tick midguts are the tissue where blood digestion occurs while salivary glands produce and secrete proteins and other molecules that modulate host defenses to enhance blood feeding [15–21]. Additionally, tick midguts and salivary glands play a major role during pathogen acquisition, multiplication and transmission [22, 23]. The functional role of these tissues during tick feeding and pathogen infection is reflected at the transcriptome and proteome levels, with tissue-specific differences between midguts and salivary glands [12, 20, 21].

Vertebrate host proteins are found in tick midguts after feeding and have been described in the salivary glands and saliva of fed ticks [24–30]. Additionally, vertebrate host proteins and particularly alpha and beta-globin chains of hemoglobin have been identified in unfed *I. scapularis* and *Amblyomma americanum* nymphs [31] and *Rhipicephalus sanguineus* adult ticks [32]. Wickramasekara et al. [31] suggested that because blood meal digestion in ticks occurs gradually within midgut cells after endocytosis [19], the combination of slow assimilation and uptake of some host proteins into tick hemolymph might explain the persistence of host blood proteins for months after feeding and molting. In fact, Francischetti et al. [33] did not identify vertebrate host proteins in salivary glands from fed soft ticks *Ornithodoros coriaceus*, probably associated with the fact that hard ticks feed for several days while soft ticks feed for less than 1 hour, therefore decreasing the probability for host proteins to persist. Nevertheless, Diaz-Martin et al. [26] did find host proteins in the saliva of the soft ticks *Ornithodoros moubata*.

These results suggest a mechanism by which host proteins remain in the tick after blood digestion. For example, host proteins persisting in the tick after molting may serve as a reserve for nutrients until the next infestation and feeding cycle are completed. However, preliminary results in questing *R. sanguineus* infected with *Rickettsia conorii* suggested that pathogen infection modify tick digestion processes, thus provoking an increase in the concentration of some host proteins such as hemoglobin ingested with blood meal in infected ticks when compared to uninfected controls [32]. These results led to the hypothesis that pathogen infection affect tick metabolic processes to modify host protein digestion and persistence in the tick, with possible implications for tick physiology and pathogen life-cycle.

To address this hypothesis, in this study we used *I. scapularis* female ticks fed on uninfected and *A. phagocytophilum*-infected sheep to characterize host proteins present in midguts and salivary glands by mass spectrometry (MS) analysis of the proteome. The results evidenced a clear difference in the sheep host protein content between tick midguts and salivary glands in response to infection and provided evidence to support that *A. phagocytophilum* selectively manipulates the levels of vertebrate host proteins in the tick vector *I. scapularis*.

## Methods

### Ethics statement

Animals were housed and experiments conducted with the approval and supervision of the Oklahoma State University Institutional Animal Care and Use Committee (Animal Care and Use Protocol, ACUP No. VM1026).

### Ticks and sample preparation

Ticks and sample preparation were previously described [12]. Briefly, *I. scapularis* ticks were obtained from the laboratory colony maintained at the Oklahoma State University Tick Rearing Facility [34]. Adult female *I. scapularis* were infected with *A. phagocytophilum* by feeding on a sheep inoculated intravenously with approximately 1 × 10^7^*A. phagocytophilum* (NY18 isolate)-infected HL-60 cells (90–100 % infected cells). In this model, over 85 % of the ticks become infected with *A. phagocytophilum* in nymphs, midguts and salivary glands. One hundred adult female ticks were removed from the sheep 7 days after infestation, held in the humidity chamber for 4 days and dissected for DNA, RNA and protein extraction from midguts and salivary glands using the AllPrep DNA/RNA/Protein Mini Kit (Qiagen, Valencia, CA, USA). Midguts and salivary glands were washed in PBS after collection to remove hemolymph-related cells. Uninfected ticks were prepared in a similar way but feeding on an uninfected sheep. Two independent samples were collected and processed for proteomics analysis for each tick tissue. Ten individual female ticks were dissected and samples collected and processed as described above to characterize *A. phagocytophilum* infection and the mRNA or protein levels of selected genes/proteins after RNA sequencing (RNAseq) or proteomics analyses.

### Proteomics data collection and analysis

Proteins were digested using the filter aided sample preparation (FASP) protocol [35]. The FASP method allows processing total SDS lysates of essentially any class of protein from biological samples of any origin, thus solving the long-standing problem of efficient and unbiased solubilization of all cellular proteins irrespective of their subcellular localization and molecular weight. Samples were dissolved in 50 mM Tris-HCl pH8.5, 4 % SDS and 50 mM DTT, boiled for 10 min and centrifuged. Protein concentration in the supernatant was measured by the Direct Detect system (Millipore, Billerica, MA, USA). About 150 μg of protein were diluted in 8 M urea in 0.1 M Tris-HCl (pH 8.5) (UA), and loaded onto 30 kDa centrifugal filter devices (FASP Protein Digestion Kit, Expedeon, TN, USA). With this method, the sample is solubilized in 4 % SDS, then retained and concentrated into microliter volumes in an ultrafiltration device. The filter unit then acts as a ‘proteomic reactor’ for detergent removal, buffer exchange, chemical modification and protein digestion. Notably, during peptide elution, the filter retains high-molecular-weight substances that would otherwise interfere with subsequent peptide separation [35]. The denaturation buffer was replaced by washing three times with UA. Proteins were later alkylated using 50 mM iodoacetamide in UA for 20 min in the dark, and the excess of alkylation reagents were eliminated by washing three times with UA and three additional times with 50 mM ammonium bicarbonate. Proteins were digested overnight at 37 °C with modified trypsin (Promega, Madison, WI, USA) in 50 mM ammonium bicarbonate at 40:1 protein:trypsin (w/w) ratio. The resulting peptides were eluted by centrifugation with 50 mM ammonium bicarbonate (twice) and 0.5 M sodium chloride. Trifluoroacetic acid (TFA) was added to a final concentration of 1 % and the peptides were finally desalted onto C18 Oasis-HLB cartridges and dried-down for further analysis. For stable isobaric labeling, the resulting tryptic peptides were dissolved in Triethylammonium bicarbonate (TEAB) buffer and labeled using the 4-plex iTRAQ Reagents Multiplex Kit (Applied Biosystems, Foster City, CA, USA) according to manufacturer’s protocol. Briefly, each peptide solution was independently labeled at room temperature for 1 h with one iTRAQ reagent vial (mass tag 114, 115, 116 or 117) previously reconstituted with 70 μl of ethanol. Reaction was stopped after incubation at room temperature for 15 min with diluted TFA, and peptides were combined. Samples were evaporated in a Speed Vac, desalted onto C18 Oasis-HLB cartridges and dried-down for further analysis. Labeled peptides were loaded into the liquid chromatography (LC)-MS/MS system for on-line desalting onto C18 cartridges and analyzing by LC-MS/MS using a C-18 reversed phase nano-column (75 μm I.D. × 50 cm, 2 μm particle size, Acclaim PepMap 100 C18; Thermo Fisher Scientific, Waltham, MA, USA) in a continuous acetonitrile gradient consisting of 0–30 % B in 145 min, 30–43 % B in 5 min and 43–90 % B in 1 min (A = 0.5 % formic acid; B = 90 % acetonitrile, 0.5 % formic acid). A flow rate of *c.*200 nl/min was used to elute peptides from the reverse phase nano-column to an emitter nanospray needle for real time ionization and peptide fragmentation on a QExactive mass spectrometer (Thermo Fisher Scientific). For increasing proteome coverage, iTRAQ-labeled samples were run at least twice. For peptide identification, all spectra were analyzed with Proteome Discoverer (version 1.4.0.29, Thermo Fisher Scientific) using a Uniprot databases containing all sequences from Ruminantia and Ixodida (April 14, 2014). For database searching, parameters were selected as follows: trypsin digestion with 2 maximum missed cleavage sites, precursor and fragment mass tolerances of 2 Da and 0.02 Da, carbamidomethyl cysteine as fixed modification and methionine oxidation as dynamic modifications. For iTRAQ labeled peptides, N-terminal and Lys iTRAQ modification was added as a fixed modification. Peptide identification was validated using the probability ratio method [36] and false discovery rate (FDR) was calculated using inverted databases and the refined method [37] with an additional filtering for precursor mass tolerance of 12 ppm. Only peptides with a confidence of at least 99 % were used to quantify the relative abundance of each peptide determined as described previously [38]. Protein quantification from reporter ion intensities and statistical analysis of quantitative data were performed as described previously using QuiXoT [39, 40]. The intensity of the reporter peaks was used to calculate the fitting weight of each spectrum in the statistical model as described previously [39]. Outliers at the scan and peptide levels and significant protein-abundance changes were detected from the *Z*-values (the standardized variable used by the model that expresses the quantitative values in units of standard deviation) by using a FDR threshold of 1 % as described previously [39]. Results are the mean of two replicates. The gene ontology (GO) analysis was performed using Uniprot (http://www.uniprot.org) annotations.

### Characterization of the digestion of sheep host hemoglobins in tick midguts and salivary glands

Sheep hemoglobin alpha 1/2 (P68240) and beta (P02075) peptides detected by MS analysis with 1 % FDR in midguts and salivary glands from uninfected and *A. phagocytophilum*-infected ticks were used for analysis. For this analysis, a new database search of MS spectra was performed with the same parameters described above but selecting “no enzyme” instead of “trypsin” digestion to identify the non-tryptic peptides present in the samples. The preferred cleavage sites for hemoglobinolytic enzymes Trypsin, Leucine aminopeptidase, Legumain, Cathepsin B, Cathepsin C, Serine carboxypeptidase were determined by searching against the MEROPS Peptidase Database (https://merops.sanger.ac.uk, release 10.0) (see in Additional file 1: Dataset S1).

### Characterization of the *I. scapularis* proteolytic and heme transport pathways mRNA and protein levels in response to *A. phagocytophilum* infection

The quantitative transcriptomics and proteomics data for midguts and salivary glands from uninfected and *A. phagocytophilum*-infected *I. scapularis* were obtained from previously published results and deposited at the Dryad Digital Repository database with the dataset identifier http://dx.doi.org//10.5061/dryad.50kt0 [12]. The analysis of the tick proteolytic pathway included the genes/proteins annotated as protease, proteinase, peptidase, and its inhibitors [41].

### Determination of hemoglobin protein levels by ELISA

Proteins were extracted from midguts and salivary glands dissected from individual uninfected and *A. phagocytophilum*-infected *I. scapularis* female ticks using the AllPrep DNA/RNA/Protein Mini Kit (Qiagen, Inc. Valencia, CA, USA) according to manufacturer instructions. Extracted proteins were resuspended in PBS with 0.5 % Triton X-100 and protein concentration was determined with the Pierce BCA Protein Assay Kit (Thermo Scientific, San Jose, CA, USA) using bovine serum albumin (BSA) as standard. Hemoglobin protein levels were determined by ELISA (Cloud-Clone Corp., Houston, TX, USA) following manufacturer instructions. Optical density values were converted to μg/ml hemoglobin using the assay standard curve and regression analysis. Hemoglobin values were compared between groups by one-tailed Student’s *t*-test for samples with unequal variance (*P* < 0.05; *n* = 2 biological replicates).

### Determination of tick mRNA levels by real-time RT-PCR

The expression of selected genes was characterized using total RNA extracted from individual *I. scapularis* female midguts and salivary glands obtained from uninfected and *A. phagocytophilum*-infected samples as previously described [12]. All ticks were confirmed as infected or uninfected by real-time PCR analysis of *A. phagocytophilum msp4* DNA. Real-time RT-PCR was performed on RNA samples with gene-specific oligonucleotide primers (see Additional file 2: Table S1) using the iScript One-Step RT-PCR Kit with SYBR Green and the iQ5 thermal cycler (Bio-Rad, Hercules, CA, USA) following the manufacturer’s recommendations. A dissociation curve was run at the end of the reaction to ensure that only one amplicon was formed and that the amplicons denatured consistently in the same temperature range for every sample. The mRNA levels were normalized against tick cyclophilin and ribosomal protein S4 as described previously using the genNorm method (ddCT method as implemented by Bio-Rad iQ5 Standard Edition, Version 2.0) [12]. Normalized Ct values were compared between infected and uninfected tick samples by Student’s *t*-test with unequal variance (*P* < 0.05; *n* = 3–17 biological replicates).

### Immunofluorescence assay (IFA)

Female ticks fed on *A. phagocytophilum*-infected and uninfected sheep and fixed with 4 % paraformaldehyde in 0.2 M sodium cacodylate buffer were embedded in paraffin and used to prepare sections on glass slides as previously described [12]. The paraffin was removed from the sections through two washes in xylene and the sections were hydrated by successive 5 min washes with a graded series of 100 %, 96 and 65 % ethanol and finally with distilled water. Next, the slides were treated with Proteinase K (Dako, Barcelona, Spain) for 7 min, washed with 0.1 % PBS-Tween 20 (Sigma-Aldrich, St. Louis, MI, USA) and blocked with 2 % bovine serum albumin (BSA; Sigma-Aldrich) in PBS-Tween 20 during 1 h at room temperature. The slides were then incubated overnight at 4 °C with rabbit anti-Cathepsin L (mature region No. pab0213-0; Covalab, Villeurbanne, France) antibodies diluted 1:1000 in 2 % BSA/PBS-Tween 20. This antibody was previously shown to recognize tick Cathepsin L by Western blot [42]. After 3 washes with PBS-Tween 20, the slides were incubated for 1 h with goat-anti-rabbit IgG conjugated with FITC (Sigma-Aldrich) diluted 1:160 in 2 % BSA/PBS-Tween 20. Finally, after two washes with PBS the slides were mounted on ProLong Diamond Antifade Mountant with DAPI reagent (Thermo Scientific™, Madrid, Spain). The sections were examined using a Leica SP2 laser scanning confocal microscope (Leica, Wetzlar, Germany) and IgGs from rabbit preimmune serum were used as controls.

### Proteomics data

Data are available via Peptide Atlas (http://www.peptideatlas.org) with identifier PASS00854.

## Results

### The vertebrate host protein content differs between tick midguts and salivary glands in response to *A. phagocytophilum* infection

Infection with *A. phagocytophilum* affects gene expression and protein production in ticks in a tissue-specific manner, but the effect on host protein content in different tick tissues has not been characterized. To address this question, sheep host proteins present in midguts and salivary glands were characterized in uninfected and *A. phagocytophilum*-infected *I. scapularis* female ticks. A total of 1,753 sheep host proteins were identified in fed adult female ticks, of which 473 were identified with more than one peptide per protein in at least one of the samples (see Additional file 2: Table S1). Of these, 1,151 (364 identified with more than one peptide per protein) and 1,282 (414 identified with more than one peptide per protein) proteins were identified in tick midguts and salivary glands, respectively (see Additional file 3: Dataset S2). Of the host proteins identified with more than one peptide per protein, 388 proteins were found in both midguts and salivary glands (see Additional file 3: Dataset S2).

Sheep host proteins showing statistically significant protein abundance changes on the basis of Zq, or standardized log2-ratio of *A. phagocytophilum-*infected versus non-infected samples (Zq > 2, Zq < −2), were selected among proteins identified with more than one peptide in at least one of the samples [12, 39]. A total of 48 (6 underrepresented and 42 overrepresented) and 50 (36 underrepresented and 14 overrepresented) differentially represented sheep host proteins were found in tick midguts and salivary glands, respectively (see Additional file 3: Dataset S2). Of them, only 8 proteins were found in both midguts and salivary glands.

**Fig. 1:**
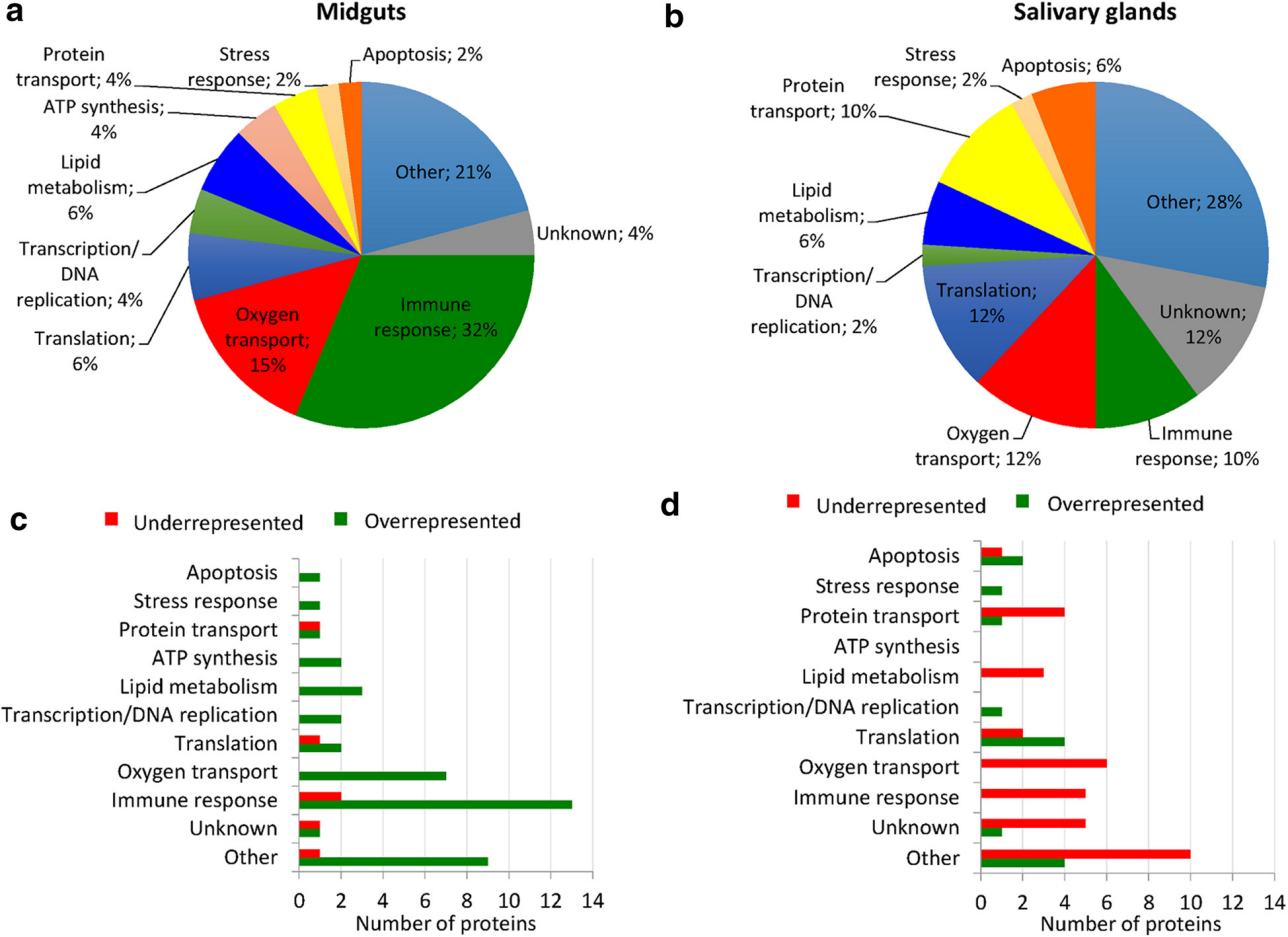
Tissue-specific effect of *A. phagocytophilum* infection on sheep host proteins represented in ticks. The results demonstrated a clear difference in the sheep host protein content between tick midguts and salivary glands in response to *A. phagocytophilum* infection. **a** Biological processes of differentially represented sheep host proteins in infected female tick midguts. **b** Biological processes of differentially represented sheep host proteins in infected female tick salivary glands. **c** Number of underrepresented and overrepresented sheep host proteins in different biological processes in infected female tick midguts when compared to uninfected controls. **d** Number of underrepresented and overrepresented sheep host proteins in different biological processes in infected female tick salivary glands when compared to uninfected controls

The GO analysis of differentially represented sheep host proteins showed that most biological processes (BPs) were found in both tick midguts and salivary glands (Fig. 1a, b). However, immune response, other and oxygen transport BPs contained 68 % of the differentially represented sheep host proteins in tick midguts while in salivary glands the most represented BPs were other, unknown, oxygen transport and translation, containing 64 % of the differentially represented sheep host proteins (Fig. 1a, b). These results evidenced a clear difference in the host protein content between tick midguts and salivary glands in response to *A. phagocytophilum* infection. Additionally, although the total number of differentially represented sheep host proteins was similar between tick tissues, 88 % of the differentially represented host proteins in midguts were overrepresented while in salivary glands only 28 % of the differentially represented proteins were overrepresented in infected ticks when compared to uninfected controls (Fig. 1c, d). Furthermore, while sheep host stress response and transcription/DNA replication proteins were overrepresented in midguts and salivary glands, proteins in the lipid metabolism, oxygen transport and immune response BPs were overrepresented in midguts and underrepresented in salivary glands in response to *A. phagocytophilum* infection (Fig. 1c, d).

### Sheep host heat shock and chromatin-related proteins are overrepresented in response to *A. phagocytophilum* infection in tick midguts and salivary glands

To characterize the putative physiological role of the host proteins differentially represented in tick midguts and salivary glands in response to *A. phagocytophilum* infection, we first focused on sheep host stress response and transcription/DNA replication proteins that were overrepresented in both tick tissues (Fig. 1c, d). The results showed that two sheep heat shock proteins (HSPs), HSP60 and HSP70, were overrepresented in infected tick midguts and salivary glands, respectively when compared to uninfected controls (Table 1). In the transcription/DNA replication BP, three host proteins involved in chromatin structure and function were overrepresented in response to *A. phagocytophilum* infection in tick midguts and salivary glands (Table 1).

**Table 1:** Sheep host stress response, transcription/DNA replication, lipid metabolism, and immune response proteins differentially represented in tick midguts and salivary glands in response to *A. phagocytophilum* infection

ID	Description	Log2 (infected/uninfected) fold change		Function
		Midguts	Salivary glands	
Stress response proteins				
P31081	HSP60	+2.9	ns	Response to cold
P0CB32	HSP70	ns	+1.4	Heat shock response
Transcription/DNA replication				
P62803	Histone H4	+3.1	ns	Chromatin structure
P68432	Histone H3.1	+3.1	ns	Chromatin structure
F1MN93	TOP1 uncharacterized protein	ns	+2.7	Chromatin binding
Lipid metabolism				
W5QHX9	Phospholipase B	+2.3	-1.3	Lipid absorption
Q9GL30	Phospholipase B	+1.9	ns	Lipid absorption
P15497	Apolipoprotein A-I	+1.9	ns	Cholesterol transport
Q32PF2	ATP-citrate synthase	ns	-1.5	Lipid synthesis
Q9TTS3	Acetyl-CoA carboxylase 1	ns	-1.6	Lipid synthesis
Immune response				
W5NQK9	S100A8	+3.8	ns	Innate immunity
W5NQJ0	S100A12	+3.2	ns	Innate immunity
W5NQH6	S100A9	+3.1	ns	Innate immunity
P28783	S100A9	+2.6	ns	Innate immunity
D8X187	Serpin peptidase inhibitor clade B ovalbumin member 1	+2.1	ns	Innate immunity
P62808	Histone H2B type 1	+3.4	ns	Adaptive immunity
W5PGJ7	PYD and CARD Domain-Containing uncharacterized protein	+2.9	ns	Adaptive immunity
P49928	Cathelin-related peptide SC5	+3.1	ns	Anti-bacterial immunity
P82018	Cathelicidin-2	+2.7	ns	Anti-bacterial immunity
P50415	Cathelicidin-3	+2.3	ns	Anti-bacterial immunity
P79360	Myeloid antimicrobial peptide	+2.6	ns	Anti-bacterial immunity
W5P7S6	Alpha-1-acid glycoprotein	+2.6	nf	Regulation of the immune response
W5PLV3	RAB5B uncharacterized protein	+2.4	nf	Antigen processing and presentation
W5PSQ7	Ig-like uncharacterized protein	ns	-1.3	Adaptive immunity
G5E513	Ig-like uncharacterized protein	ns	-1.8	Adaptive immunity
G5E5T5	Ig-like uncharacterized protein	ns	-2.7	Adaptive immunity
F1MQF6	Apoptosis-associated speck-like protein-containing a CARD	nf	-1.5	Innate immunity
W5PGJ7	LOC101105208 uncharacterized protein	ns	-1.6	Anti-bacterial immunity

*Abbreviations*: *ID* protein (Uniprot; http://www.uniprot.org) accession numbers; +, overrepresented proteins in infected *vs* uninfected ticks; -, underrepresented proteins in infected *vs* uninfected ticks; nf, not found; ns, not significant

### Sheep host proteins involved in lipid metabolism and immune response are overrepresented in tick midguts but underrepresented in salivary glands in response to *A. phagocytophilum* infection

To characterize further the putative physiological role of the host proteins differentially represented in response to *A. phagocytophilum* infection, we then focused on sheep host proteins in the lipid metabolism and immune response BPs that were overrepresented in midguts and underrepresented in salivary glands in response to *A. phagocytophilum* infection (Fig. 1c, d). The host lipid metabolism proteins overrepresented in *A. phagocytophilum*-infected tick midguts when compared to uninfected controls included proteins involved in lipid absorption, transport and excretion (Table 1). In tick salivary glands, sheep host proteins involved in lipid synthesis were underrepresented in infected ticks when compared to uninfected controls (Table 1).

In tick midguts, sheep host immune response proteins that were overrepresented in response to *A. phagocytophilum* infection included proteins involved in innate immunity (including several S100 proteins), adaptive immunity, anti-bacterial immunity, regulation of the immune response, and antigen processing and presentation (Table 1). In tick salivary glands, sheep host immunoglobulin (Ig)-like proteins and proteins involved in innate and anti-bacterial immunity were underrepresented in response to *A. phagocytophilum* infection (Table 1).

To confirm the origin of selected differentially represented proteins (Table 1), all of the peptides used to identify the proteins sharing tryptic peptides with *I. scapularis* proteins were revised to show the sequence of the peptides that are exclusive for host-derived proteins (Table 2).

**Table 2:** Identification of host-derived proteins with identical tryptic peptides to *I. scapularis* tick homologues

ID	Description	Unique host-derived peptides
P31081	HSP60	ALMLQGVDLLADAVAVTMGPK
		VGGTSDVEVNEK
		VGGTSDVEVNEKKDR
P0CB32	HSP70	FDLTGIPPAPR
		RKELEQVCNPIITK
P68432	Histone H3.1	RVTIMPKDIQLAR
		SAPATGGVK
		SAPATGGVKKPHRYRPGTVALR
F1MN93	TOP1 uncharacterized protein	AGNEKEEGETADTVGCCSLR
		HLQDLMEGLTAK
P62808	Histone H2B type 1	AMGIMNSFVNDIFER
		EIQTAVRLLLPGELAK
		EIQTAVR
		ESYSVYVYK
		SRKESYSVYVYK
		STITSREIQTAVRLLLPGELAK
		STITSREIQTAVR
		VLKQVHPDTGISSK
W5PLV3	RAB5B uncharacterized protein	TAMNVNDLFLAIAK
P62803	Histone H4	None
Q9TTS3	Acetyl-CoA carboxylase 1	None

To confirm the origin for selected differentially represented proteins (Table 1), all of the peptides used to identify the proteins sharing tryptic peptides with *I. scapularis* proteins were revised. The peptides unique for host-derived proteins are shown. For protein P62803, we could not define the origin due to 100 % homology between sheep and tick proteins. For protein Q9TTS3, all peptides used for identification were identical in both host and tick-derived proteins

### *Anaplasma phagocytophilum* infection impacts on vertebrate host hemoglobin content in tick midguts and salivary glands

**Fig. 2:**
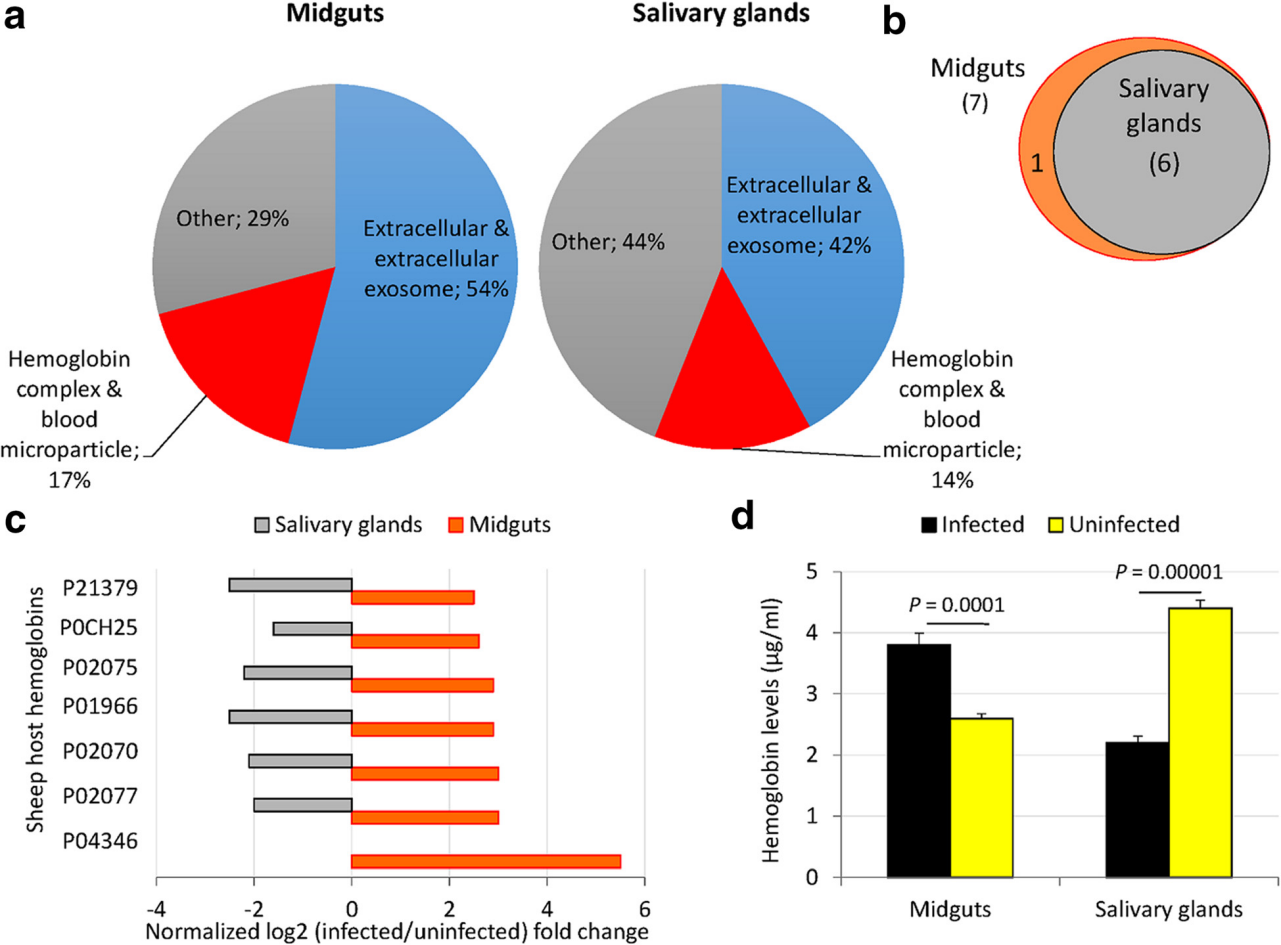
Sheep host hemoglobin levels vary in a tissue-specific manner in response to *A. phagocytophilum* infection in ticks. **a** Cell compartment classification of differentially represented sheep host proteins in infected female tick midguts and salivary glands. **b** Venn diagram of the sheep host hemoglobin differentially represented in infected *vs* uninfected tick tissues. **c** Differential host hemoglobin protein representation in response to *A. phagocytophilum* infection in tick midguts and salivary glands. **d** Hemoglobin levels in tick midguts and salivary glands from *A. phagocytophilum*-infected and uninfected ticks determined by ELISA in individual tick protein extracts, represented as the mean + standard deviation (SD) and compared between samples from infected and uninfected ticks by Student’s *t*-test with unequal variance (*P* < 0.05; 2 biological replicates)

The identification of differentially represented sheep host proteins in *A. phagocytophilum*-infected *I. scapularis* midguts and salivary glands suggested the question about the origin of these proteins. The analysis of cell compartment GO showed that over 50 % of the proteins were extracellular or associated with blood cells (Fig. 2a; see Additional file 3: Dataset S2). Nevertheless, other proteins were localized in the cell cytoplasm (Fig. 2a; see Additional file 3: Dataset S2), probably associated with host blood cells ingested by ticks during feeding. Most of the host proteins in the hemoglobin complex and blood microparticle classification were sheep hemoglobins in the oxygen transport BP represented in both tick midguts and salivary glands (Fig. 2b). These hemoglobins were overrepresented in midguts and underrepresented in salivary glands of *A. phagocytophilum*-infected ticks when compared to uninfected controls (Fig. 2c), a result that was corroborated by an independent analysis using a specific ELISA test (Fig. 2d).

### *Anaplasma phagocytophilum* manipulates host protein content through modification of tick proteolytic pathways

**Fig. 3:**
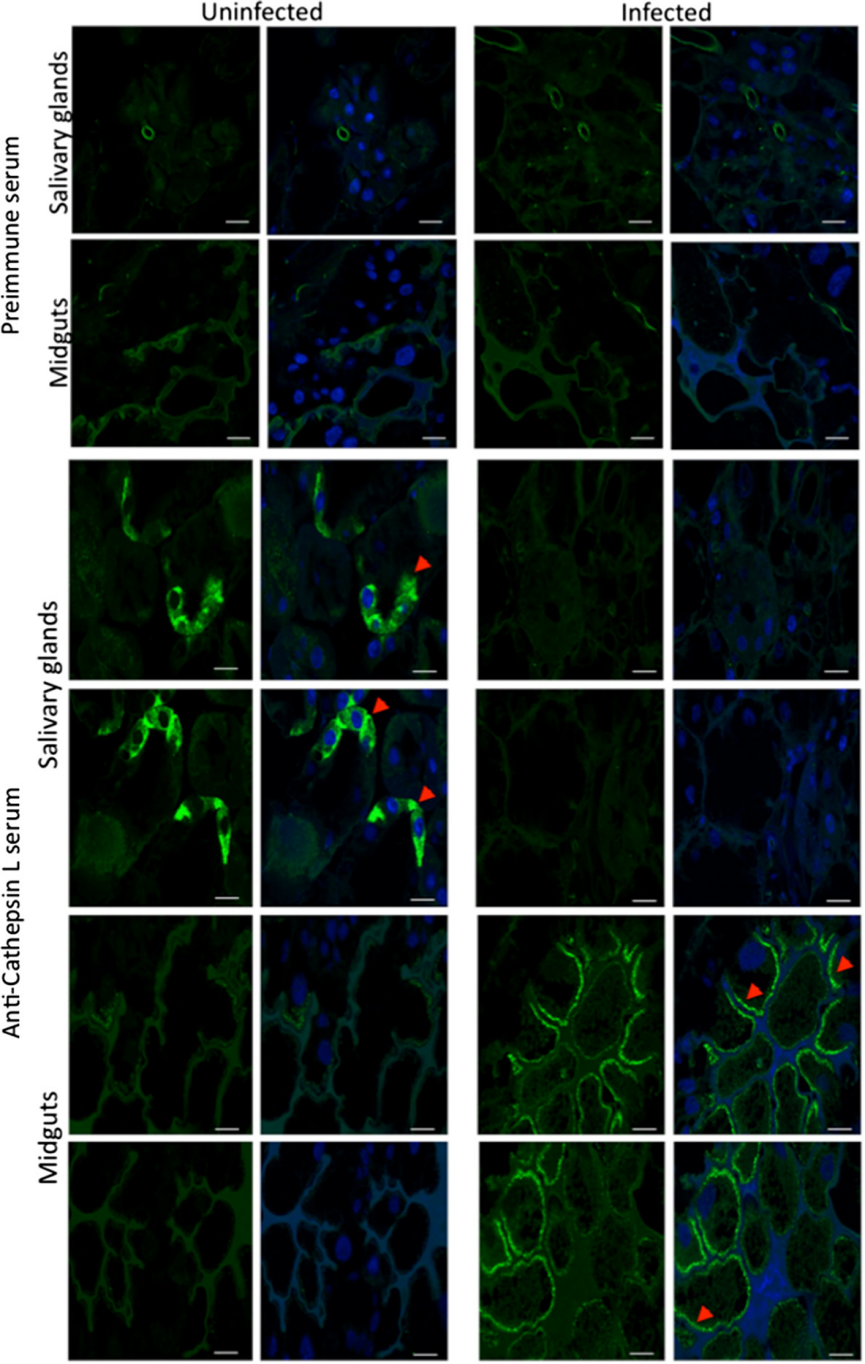
Characterization of Cathepsin L protein levels by IFA. Representative images of IFA of midguts and salivary glands of uninfected and *A. phagocytophilum*-infected adult female *I. scapularis*. Tick tissues were stained with rabbit anti-Cathepsin L (mature region No. pab0213-0; Covalab, Villeurbanne, France) antibodies (*green*, FITC) or DAPI (*blue*), and images were superimposed after staining (right panels). Preimmune control serum-treated samples showed similar results for uninfected and infected ticks. Uninfected and infected samples stained with anti-Cathepsin L antibodies showed higher protein levels in infected midguts while Cathepsin L was underrepresented in infected salivary glands when compared to uninfected controls (arrowheads). *Scale-bars*: 10 μm

To aid in the probable mechanism responsible for the differential representation of host proteins in midguts and salivary glands of *A. phagocytophilum*-infected ticks when compared to uninfected controls, the tissue-specific effect of infection was characterized on tick hemoglobinolytic enzymes and other proteases. The transcriptomics and proteomics data used in this study was previously validated by real-time RT-PCR and Western blot or immunofluorescence for selected genes and proteins, respectively [12, 43]. Nevertheless, 5 selected genes coding for hemoglobin digesting enzymes differentially regulated in response to *A. phagocytophilum* infection were used for analysis by real-time RT-PCR in individual tick midguts and salivary glands (see Additional file 2: Figure S1). As previously discussed [12], the differences observed between the results of both analyses that were evident in tick midguts considering the absence of transcriptomics data for some genes in salivary glands, could be attributed to intrinsic variation in gene expression and the fact that approximately 85 % of the ticks used for RNAseq were infected [44], while for real-time RT-PCR all ticks were confirmed uninfected or infected with *A. phagocytophilum* before analysis. At the protein level, an antibody recognizing tick Cathepsin L was used to corroborate proteomics results by IFA. Similar to proteomics analysis (Table 3), the results showed protein underrepresentation in the salivary glands of *A. phagocytophilum*-infected ticks when compared to uninfected controls (Fig. 3). Furthermore, although proteomics data were not available, Cathepsin L was overrepresented in tick midguts in response to infection (Fig. 3). Therefore, considering these results, the analysis of the differential expression/representation of tick hemoglobinolytic enzymes in response to *A. phagocytophilum* infection was presented by pondering mRNA (transcriptomics RNAseq and real-time RT-PCR) and protein (proteomics) data (see Additional file 2: Figure S2).

**Table 3:** Differential expression/representation of enzymes involved in tick hemoglobinolytic and heme transport pathways in response to *A. phagocytophilum* infection

ID	Description	Log2 (infected/uninfected) fold change (mRNA/protein)		Role in hemoglobin digestion
		Midguts	Salivary glands	
Tick hemoglobinolytic pathway				
EF428204	Cathepsin D	nf/ns	ns/nf	Primary cleavage
A4GTA5				
HQ615697	Cathepsin D2	nf/ns	ns/nf	Primary cleavage
E7E820				
ISCW000202	Legumain	-0.6/ns	ns/ns	Primary cleavage
B7P6S9				
ISCW015983	Legumain	-1.3/nf	nf/nf	Primary cleavage
B7P2C6				
ISCW000076	Cathepsin L	-7.3/nf	nf/nf	Primary and secondary cleavage
B7P3N8				
JX502821	Cathepsin L	nf/nf	nf/-2.9	Primary and secondary cleavage
J9QSA1				
ISCW000080	Cathepsin B	+0.5/ns	nf/-2.1	Secondary and tertiary cleavage
B7P3P1				
EU551624	Cathepsin B	nf/ns	nf/-2.0	Secondary and tertiary cleavage
B7SP39				
ISCW013346	Cathepsin B	-1.6/ns	ns/-2.5	Secondary and tertiary cleavage
B7QCU7				
ISCW000078	Cathepsin B	+0.2/ns	-4.3/-1.8	Secondary and tertiary cleavage
B7P3P0				
ISCW003494	Cathepsin C	+0.3/ns	-2.6/+2.2	Tertiary cleavage
B7PEB4				
ISCW001779	Leucine aminopeptidase	+2.2/ns	ns/nf	Tertiary cleavage
B7P2N4				
ISCW023735	Leucine aminopeptidase	+2.0/ns	+1.1/ns	Tertiary cleavage
B7QLQ7				
ISCW001780	Leucine aminopeptidase	+1.4/ns	+0.5/ns	Tertiary cleavage
B7P2N5				
ISCW013904	Serine carboxipeptidase	+1.0/ns	ns/ns	Tertiary cleavage
B7QLB7				
ISCW024536	Serine carboxipeptidase	-0.5/ns	ns/nf	Tertiary cleavage
B7Q049				
ISCW024751	Serine carboxipeptidase	-0.7/ns	ns/ns	Tertiary cleavage
B7QD81				
ISCW024883	Serine carboxipeptidase	-4.5/nf	-2.4/nf	Tertiary cleavage
B7QK83				
ISCW007492	Serine carboxipeptidase	-1.7/ns	ns/ns	Tertiary cleavage
B7PTE5				
ISCW003059	Serine carboxipeptidase	-1.2/nf	ns/nf	Tertiary cleavage
B7PC00				
Tick heme transport pathway				
ISCW001847	Heme-responsive gene 1 (HRG1)	-0.8/nf	ns/nf	Heme transporter
B7P8M4				
ISCW021709	Heme-binding lipoprotein (HELP)	+3.3/ns	-0.2/ns	Heme transporter
B7Q406				
ISCW013727	Vitellogenin 1 (VG1)	ns/ns	ns/nf	Heme transporter
B7QJ67				
ISCW021228	Vitellogenin 2 (VG2)	-1.1/nf	ns/nf	Heme transporter
B7Q7E5				

Transcriptomics RNAseq and proteomics data from *A. phagocytophilum*-infected and uninfected tick samples were obtained from Ayllón et al. [12]. Except for Cathepsins D and D2, which were included to show that these proteins were identified in the proteomics analysis but were not significantly different between infected and uninfected samples, only genes/proteins with statistically significant differences in at least one of the analyses (transcriptomics or proteomics) and samples (midguts or salivary glands) were included. *Abbreviations*: ID, gene (GenBank; http://www.ncbi.nlm.nih.gov) and protein (Uniprot; http://www.uniprot.org) accession numbers

+, upregulated/overrepresented genes/proteins in infected *vs* uninfected ticks; -, downregulated/underrepresented genes/proteins in infected *vs* uninfected ticks; nf, not found; ns, not significant

**Fig. 4:**
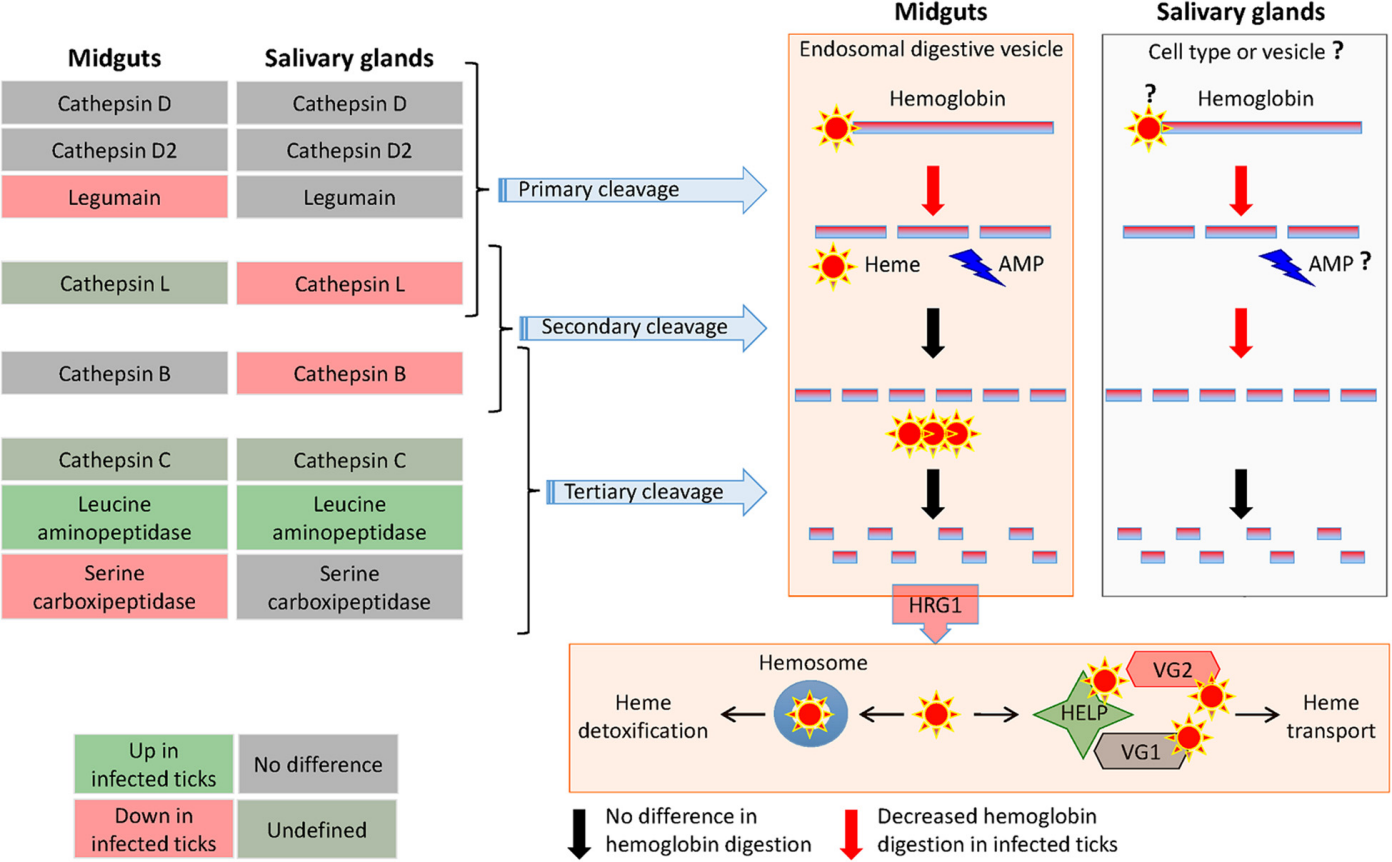
The levels of enzymes involved in the tick hemoglobinolytic pathway vary in a tissue-specific manner in response to *A. phagocytophilum* infection. Differential expression/representation of tick hemoglobinolytic enzymes in response to *A. phagocytophilum* infection was obtained from Ayllón et al. [12] and represented by pondering mRNA (transcriptomics RNAseq and real-time RT-PCR) and protein (proteomics) data. In tick midguts, the hemoglobinolytic pathway operating in the endosomal digestive vesicle was revised by Sojka et al. [19]. In tick salivary glands, these enzymes are also produced and may function under different conditions. *Abbreviations*: AMP, hemoglobin-derived antimicrobial peptides (Hemocidins and other); HRG1, Heme-responsive gene 1; HELP, Heme-binding lipoprotein; VG1, Vitellogenin 1; VG2, Vitellogenin 2

**Fig. 5:**
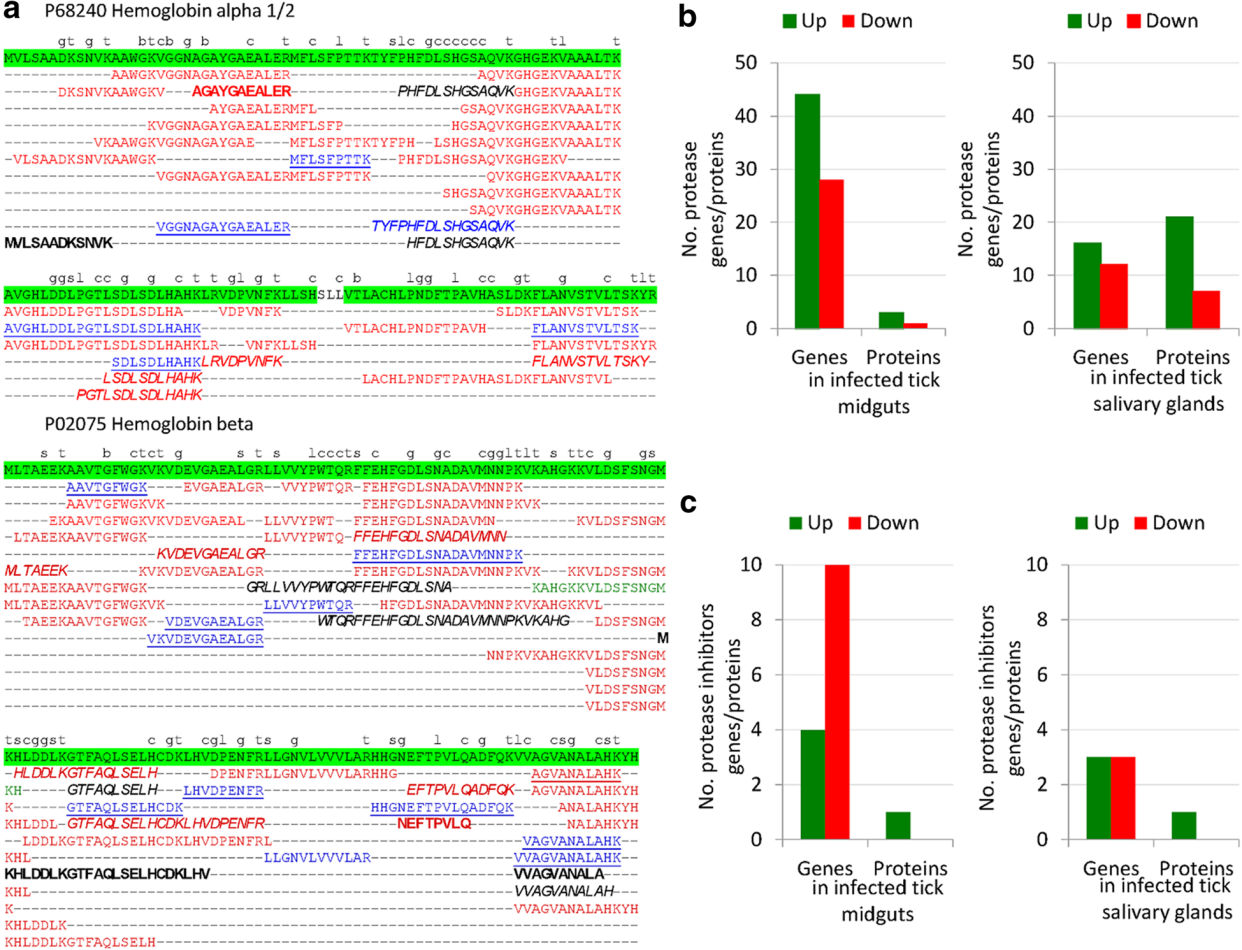
The digestion of sheep host hemoglobin varies between tick midguts and salivary glands in a tissue-specific manner in response to *A. phagocytophilum* infection. **a** Sheep hemoglobin alpha 1/2 (P68240) and beta (P02075) peptides detected by MS analysis with 1 % FDR in midguts and salivary glands from uninfected and *A. phagocytophilum*-infected ticks. Peptides detected in uninfected and infected (*blue*), infected (*red*), or uninfected (*green*) tick midguts and in uninfected and infected (*underlined*), infected (*bold*), or uninfected (*italics*) tick salivary glands are shown. Hemoglobin protein coverage by detected peptides is highlighted in *green*. The preferred cleavage sites for Trypsin and hemoglobinolytic enzymes are shown over P1 amino acid for Trypsin (t), Leucine aminopeptidase (l), Legumain (g), Cathepsin B (b), Cathepsin C (c), and Serine carboxypeptidase (s) (see Additional file 3: Dataset S2). **b** The number of protease genes/proteins different from hemoglobinolytic enzymes and differentially expressed/represented in response to *A. phagocytophilum* infection in tick midguts and salivary glands were extracted from transcriptomics and proteomics data [12]. **c** The number of protease inhibitor genes/proteins differentially expressed/represented in response to *A. phagocytophilum* infection in tick midguts and salivary glands were extracted from transcriptomics and proteomics data [12]

The results suggested that in midguts from *A. phagocytophilum*-infected ticks when compared to uninfected controls, the hemoglobin primary cleavage was inhibited after Legumain underrepresentation while hemoglobin secondary and tertiary cleavages were probably not affected (Fig. 4 and Table 3). The hemoglobinolytic enzymes were also found in tick salivary glands, suggesting a role in hemoglobin digestion in this tissue (Table 3). In the salivary glands of infected ticks when compared to uninfected controls, the results suggested that hemoglobin primary and secondary cleavages were inhibited because Cathepsins L and B were underrepresented in response to infection while the hemoglobin tertiary cleavage was probably not affected (Fig. 4 and Table 3). The analysis of sheep hemoglobin alpha 1/2 (P68240) and beta (P02075) peptides identified by MS in tick midguts and salivary glands showed the presence of potential cleavage sites for trypsin (used in protein digestion for MS analysis), Legumain, Cathepsin B, Cathepsin C, Leucine aminopeptidase and Serine carboxipeptidase, therefore providing additional support for the activity of these enzymes in both tick tissues (Fig. 5a; see Additional file 1: Dataset S1).

In the midguts of ticks infected with *A. phagocytophilum*, Heme-responsive gene 1 protein (HRG1) was underrepresented, while heme transport proteins Heme-binding lipoprotein (HELP) and Vitellogenin 2 (VG2) but not Vitellogenin 1 (VG1) were overrepresented and underrepresented, respectively, in response to infection (Fig. 4 and Table 3). Furthermore, sheep host blood coagulation factors Annexin A3 (Q3SWX7; overrepresented in infected tick midguts and involved in blood anti-coagulation as a Phospholipase 2 inhibitor), and Fibrinogen gamma-B, and uncharacterized protein APOH (P12799 and W5Q268; underrepresented in infected tick salivary glands and involved in blood coagulation) were differentially represented in infected ticks when compared to uninfected controls (see Additional file 3: Dataset S2), resulting in the inhibition of blood coagulation in both tick tissues.

In addition to hemoglobinolytic enzymes, other tick proteases were upregulated/overrepresented while protease inhibitors were down-regulated or did not change in midguts and salivary glands of *A. phagocytophilum*-infected ticks when compared to uninfected controls (Fig. 5b, c). However, as shown for the hemoglobinolytic enzymes (Fig. 3), the tick proteases differentially regulated in response to infection were predominantly different between midguts and salivary glands (see Additional file 2: Table S2).

## Discussion

The characterization of sheep host proteins in the midguts and salivary glands of uninfected and *A. phagocytophilum*-infected *I. scapularis* female ticks showed tissue-specific differences in response to infection. Vertebrate host proteins in the transcription, lipid metabolism, immune response and oxygen transport (hemoglobins) were previously found to be highly abundant in the saliva of engorged *I. scapularis* ticks [24, 30]. The authors suggested that ticks have evolved mechanisms to selectively secrete host proteins in the saliva to aid in the feeding process [30]. Furthermore, anti-microbial peptides (AMP) such as S100 proteins [45] highly abundant in the saliva of engorged *I. scapularis* were proposed to function in clearing microbes from the feeding site to preserve ticks [30]. In *A. phagocytophilum*-infected ticks, host proteins from some of these pathways such as stress response and transcription were overrepresented in tick midguts and salivary glands, supporting the existence of a mechanism to facilitate tick feeding that was enhanced in response to infection. However, other host proteins overrepresented in *A. phagocytophilum*-infected tick midguts probably reflected the host response to infection. For example, proteins in the immune response BP are upregulated at the transcriptional level in sheep infected with *A. phagocytophilum* [46]. Nevertheless, immune response proteins were underrepresented in infected tick salivary glands when compared to uninfected controls, suggesting that *A. phagocytophilum* selectively manipulates the levels of host proteins to facilitate pathogen infection, multiplication and transmission.

The infection with *A. phagocytophilum* modulates lipid metabolism in vertebrate host cells and bacteria incorporate host cholesterol for survival [47–49]. In tick cells, *A. phagocytophilum* infection inhibits lipid metabolism through down-representation of tick proteins [50]. The overrepresentation of sheep host proteins involved in lipid absorption, transport and secretion in midguts and the underrepresentation of lipid synthesis proteins in salivary glands of infected ticks when compared to uninfected controls may constitute an additional mechanism by which *A. phagocytophilum* selectively manipulates lipid metabolism to enhance infection and multiplication in tick tissues.

Although ticks contain genes that encode heme synthesis enzymes, recent results demonstrate that they do not synthesize heme but obtain heme from the vertebrate host hemoglobin in the midgut and from tick heme transporters HELP/VG1/VG2 in other tissues [50–52]. Recently, Hajdusek et al. [50] proposed that the heme produced after host hemoglobin digestion is transported outside the endosomal digestive vesicle by HRG1 and subsequently detoxified in the hemosome or transported by HELP, VG1 and VG2 to other tick tissues such as salivary glands. However, as shown here and in previous reports [30], the presence of active tick hemoglobinolytic enzymes in the salivary glands and secreted in the saliva of engorged *I. scapularis* suggests the possibility that host hemoglobin may be also digested under different conditions to provide heme in the salivary glands. Although heme may not contribute to the cellular iron pool in ticks [52], the results reported here suggested that *A. phagocytophilum* affects hemoglobin primary cleavage in tick midguts and salivary glands, probably to reduce the production of hemoglobin-derived AMP to facilitate pathogen multiplication [50]. Furthermore, although *A. phagocytophilum* infection did not affect most of the enzymes involved in hemoglobin secondary and tertiary cleavage in tick midguts, the underrepresentation of HGR1 suggested a mechanism to reduce heme release into the cytoplasm of midgut cells (Fig. 4). This mechanism is probably manipulated by *A. phagocytophilum* to facilitate infection through reduction of the antimicrobial oxidative burden caused by reactive oxygen species (ROS) generated after heme release [19, 53, 54]. Furthermore, the inhibition of blood coagulation may be a mechanism driven by tick and/or *A. phagocytophilum* to facilitate tick feeding and pathogen multiplication. As recently proposed [13], these mechanisms may have evolved to guarantee *A. phagocytophilum* infection, multiplication and transmission while preserving tick life cycle.

Once shown that *A. phagocytophilum* selectively manipulates the levels of vertebrate host proteins in ticks in a tissue-specific manner, the next question was why these proteins were selected among all host proteins ingested by ticks during blood feeding? Some of these host proteins such as hemoglobins, S100 and Ig-like proteins that were overrepresented in tick midguts and underrepresented in salivary glands have a crucial role during tick feeding and pathogen infection. In feeding ticks, host hemoglobins are the source of heme and AMP, which together with other immune system proteins such as S100 and Ig-like proteins may be essential for tick feeding and antimicrobial response to control microbe levels in tick tissues [19, 30, 45, 50, 53, 54]. Additionally, most of these proteins are highly conserved among major domestic and natural vertebrate hosts for *A. phagocytophilum* and *I. scapularis* (see Additional file 2: Table S3). Therefore, these results suggested that the mechanisms responsible for the selective manipulation of vertebrate host proteins by *A. phagocytophilum* infection in tick tissues are evolutionary conserved.

The physiological significance of these findings was addressed by responding to the questions recently proposed by Sojka et al. [19] for a better understanding of how ticks handle the blood meal. Among these questions they proposed to address if the same tick enzyme machinery process hemoglobin and other vertebrate host proteins and the role of blood digestion and chemical reduction-oxidation reaction balance on pathogen infection and transmission in the tick midgut. The results of our study showed that tick hemoglobinolytic enzymes are present and active in both midguts and salivary glands of fed ticks and therefore may be involved in the digestion of hemoglobin and other host proteins. Although the effect of hemoglobin digestion and ROS production on pathogen infection and transmission was not directly addressed in our study, the results suggested that *A. phagocytophilum* selectively manipulate these and other processes to facilitate pathogen infection, multiplication and transmission.

The results reported here suggested that the mechanism used by *A. phagocytophilum* to selectively manipulate the levels of vertebrate host proteins in a tissue-specific manner is through modification of tick proteolytic pathways. How *A. phagocytophilum* modify tick proteolytic pathways is not known, but may include the regulation of gene expression through epigenetic mechanisms recently shown to be affected by pathogen infection in *I. scapularis* [43]. These epigenetic mechanisms are probably controlled by secreted bacterial effectors [55–58]. However, future experiments should address the physiological significance of tick proteolytic pathways during *A. phagocytophilum* infection and multiplication in midguts and salivary glands.

## Conclusions

**Fig. 6:**
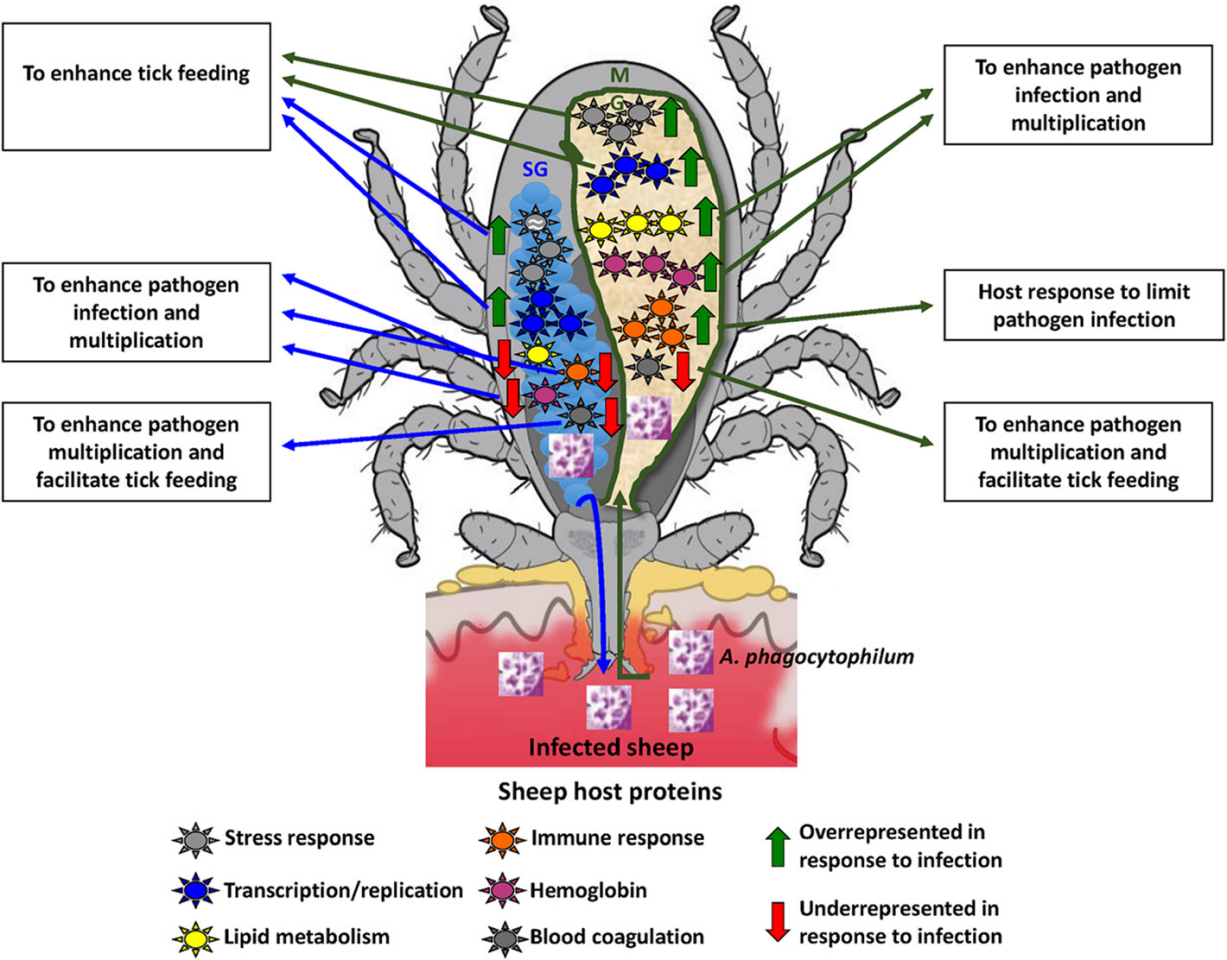
Proposed functional significance for the host proteins selectively manipulated by *A. phagocytophilum* in tick midguts and salivary glands. These results suggested that *A. phagocytophilum* selectively manipulates the levels of vertebrate host proteins in the tick midguts (MG) and salivary glands (SG) to facilitate pathogen infection, multiplication and transmission while preserving tick feeding and development

In summary, the results of this study corroborated that vertebrate host proteins are present in the midguts and salivary glands of fed female *I. scapularis*. To our knowledge, the results presented here showed for the first time that *A. phagocytophilum* selectively manipulates the levels of vertebrate host proteins in the tick vector to facilitate pathogen infection, multiplication and transmission while preserving tick feeding and development (Fig. 6). The mechanisms by which *A. phagocytophilum* manipulates the levels of vertebrate host proteins are not known, but may include modification of proteolytic pathways by affecting tick epigenetics and other biological processes.

Despite the growing burden that *A. phagocytophilum* and other tick-borne pathogens represent for human and animal health worldwide, effective control measures have not been developed [59]. Investigating the biological relevance of host proteins in tick biology and pathogen infection and the mechanisms used by *A. phagocytophilum* to manipulate host protein content is essential to advance our knowledge of tick-host-pathogen molecular interactions. These results have implications for the identification of new targets for the development of vaccines for the control of tick-borne diseases.

## Additional files

Additional file 1: Dataset S1. Digestion of sheep host hemoglobin in tick midguts and salivary glands. (XLS 55 kb) Additional file 2: Figure S1. Analysis of RNAseq results by real-time RTPCR. **Figure S2.** Pondering mRNA and protein data.** Table S1.** Genes and oligonucleotide primers selected for gene expression analysis by real-time RT-PCR. **Table S2.** Differential expression of highly differentially regulated tick protease genes in response to *A. phagocytophilum* infection. **Table S3.** Percent homology for selected differentially represented host proteins in tick midguts and salivary glands in response to *A. phagocytophilum* infection. (PDF 337 kb) Additional file 3: Dataset S2. Proteomics results for sheep host proteins in tick tissues. (XLSX 208 kb)

## Abbreviations

HGA: human granulocytic anaplasmosis
MS: mass spectrometry
RNAseq: RNA sequencing
FASP: filter aided sample preparation
TFA: trifluoroacetic acid
TEAB: TriethyLammonium bicarbonate
LC: liquid chromatography
FDR: false discovery rate
GO: gene ontology
IFA: immunofluorescence assay
BSA: bovine serum albumin
BP: biological process
HSP: heat shock protein
Ig: immunoglobulin
HRG1: Heme-responsive gene 1 protein
HELP: Heme-binding lipoprotein
VG1: Vitellogenin 1
VG2: Vitellogenin 2
ROS: reactive oxygen species
AMP: anti-microbial peptides
