# Supplementary material for: The intracellular bacterium Anaplasma phagocytophilum selectively manipulates the levels of vertebrate host proteins in the tick vector Ixodes scapularis
Source: Parasit Vectors. 2016 Aug 25;9(1):467. doi: 10.1186/s13071-016-1747-3 (PMC5000436; doi:10.1186/s13071-016-1747-3)
Supplement: Additional file 2: Figure S1. — Analysis of RNAseq results by real-time RTPCR. Figure S2. Pondering mRNA and protein data. Table S1. Genes and oligonucleotide primers selected for gene expression analysis by real-time RT-PCR. Table S2. Differential expression of highly differentially regulated tick protease genes in response to A. phagocytophilum infection. Table S3. Percent homology for selected differentially represented host proteins in tick midguts and salivary glands in response to A. phagocytophilum infection. (PDF 337 kb) [file 13071_2016_1747_MOESM2_ESM.pdf]

Supplemental Material

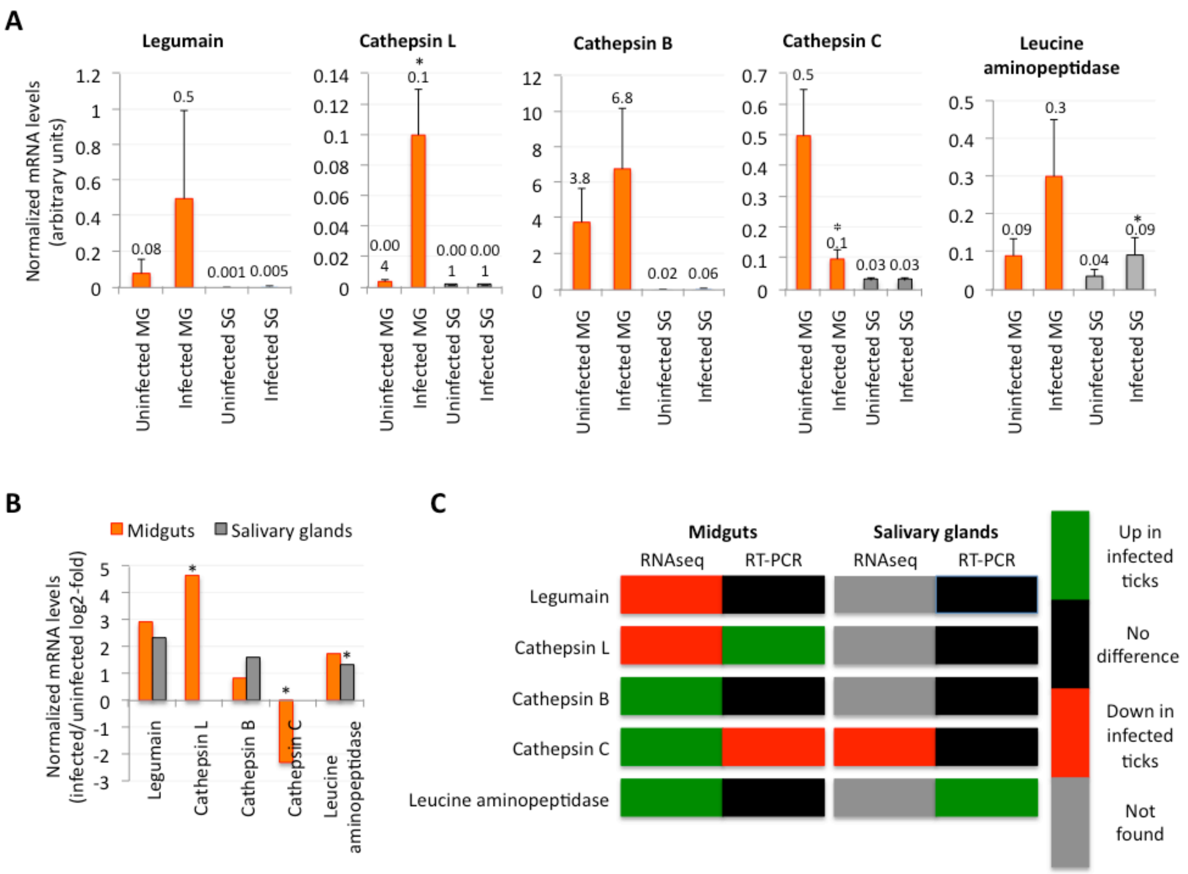

Figure S1. Analysis of RNAseq results by real-time RT-PCR. Five selected genes coding for hemoglobin digesting enzymes Legumain (ISCW015983), Cathepsin L (ISCW000076), Cathepsin B (ISCW000080), Cathepsin C (ISCW003494) and Leucine aminopeptidase (ISCW001779) differentially regulated in response to *A. phagocytophilum* infection were used for analysis by real-time RT-PCR in individual tick midguts and salivary glands. (A) The mRNA levels were normalized against tick 16S rRNA and cyclophilin, represented as Ave+SD and compared between infected and uninfected ticks by Student's t-test with unequal variance (*P≤0.05; N=3-17 biological replicates). (B) Normalized mRNA levels are represented as infected/uninfected Log2-fold ratio and compared between infected and uninfected ticks by Student's t-test with unequal variance (*P≤0.05). (C) Differential expression of selected tick genes was compared between RNAseq and real-time RT-PCR results in midguts and salivary glands.

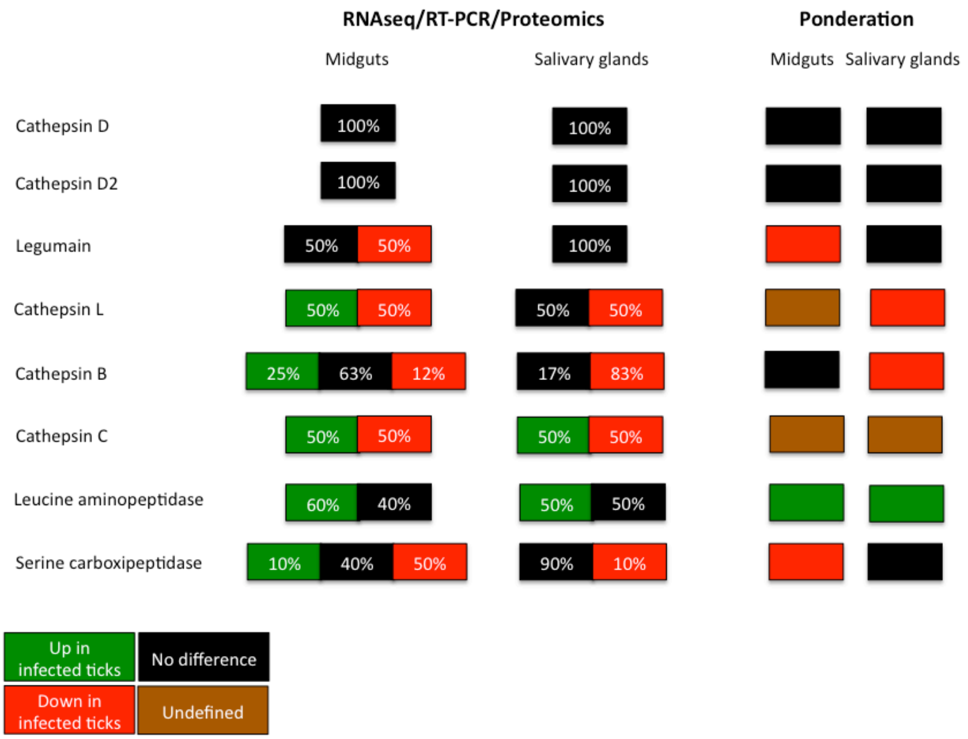

Figure S2. Pondering mRNA and protein data. Transcriptomics RNAseq data (Ayllón et al. 2015), real-time RT-PCR results obtained in this study and proteomics data (Ayllón et al. 2015) were considered when pondering the effect of A. phagocytophilum infection in the gene expression/protein representation of tick hemoglobinolytic enzymes in midguts and salivary glands. The results are shown as percent of genes/proteins for each enzyme that were up, down or without differences between infected and uninfected ticks. The final ponderation was made considering that 50% or more of the enzymes were up or down in response to infection. Otherwise, enzymes were considered that did not change or their levels could not be defined in response to infection.

Table S1. Genes and oligonucleotide primers selected for expression analysis by real-time RT-PCR.

***I.scapularis* genes**	**Genbank accession No.**	**Forward and Reverse primers for RTPCR (5´- 3´)**	**PCR annealing conditions**
Cathepsin B	ISCW000080	GGCAGGAAGGAACTTCGACA<br>CTGGTCGCGGATAAGGTGAA	60°C/30s
Cathepsin C	ISCW003494	CGAAAGACGTTCGATGCGAC<br>CACCTCGAAGCCCTGATTGT	60°C/30s
Cathepsin L	ISCW000076	CAGCGAAGGAAAGGTCCAGGT<br>TTACCCGTAACCGCAGGAATGG	63°C/30s
Legumain	ISCW015983	CCCCGGAGAACTTCCTGAAC<br>CTTGCCGAACCTGTTTTCCG	60°C/30s
Leucine aminopeptidase	ISCW001779	ATGCAGACTTCGTCCTCACG<br>CTCATCGCGGTCTTCCAGTT	60°C/30s
Cyclophilin	ISCW000776	GCTTCGGTTACAAGGGCAGCAGCATTT<br>TCGGGTGTGCTTCAGGATGAAGTT	60°C/30s
Ribosomal protein S4	DQ066214	GGTGAAGAAGATTGTCAAGCAGAG<br>TGAAGCCAGCAGGGTAGTTTG	60°C/30s

Table S2. Differential expression of highly differentially regulated tick protease genes in response to *A. phagocytophilum* infection.

ID	Description	Log2 (infected/uninfected) fold change (mRNA)
**Peptidases in tick midguts**		
ISCW004264	Carboxypeptidase*	+3.2
ISCW020713	Aminopeptidase	+2.8
ISCW003960	Dipeptidylpeptidase	+2.6
ISCW022741	Polylcarboxypeptidase*	-7.4
ISCW022742	Polylcarboxypeptidase	-6.0
ISCW022743	Polylcarboxypeptidase	-4.0
**Peptidases in tick salivary glands**		
ISCW022411	Glutamate carboxypeptidase	+3.9
ISCW007964	Carboxypeptidase	+2.9
ISCW001311	Carboxypeptidase	+1.6
ISCW022741	Polylcarboxypeptidase*	-6.3
ISCW004264	Carboxypeptidase*	-2.7
ISCW020703	Signal peptidase complex subunit SPC25	-2.1
**Proteases in tick midguts**		
ISCW003013	Sentrin/sumo-specific protease	+4.8
ISCW011212	Ubiquitin-specific protease	+3.0
ISCW001119	Serine protease	+2.8
ISCW001619	Serine protease	-3.8
ISCW014841	Secreted serine protease	-3.8
ISCW012429	Serine protease	-3.5
**Proteases in tick salivary glands**		
ISCW013599	Salivary metalloprotease	+3.5
ISCW007161	Metalloendopeptidase	+2.8
ISCW019965	Ubiquitin-specific protease	+2.2
ISCW003039	Caspase, spoptotic cysteine proease	-4.8
ISCW006994	Metalloprotease	-2.0
ISCW016405	Metalloendopeptidase	-1.4
**Proteinases in tick midguts**		
ISCW001423	Zinc metalloproteinase	+4.0
ISCW007768	Serine proteinase	+2.8
ISCW014005	Serine proteinase	+2.3
ISCW006136	Matrix metalloproteinase	-2.9
ISCW015172	Serine proteinase	-2.5
ISCW012738	Midgut serine proteinase 1	-2.0
**Proteinases in tick salivary glands**		
ISCW007716	Ubiquitin-specific proteinase	+3.4

Proteases included in the analysis correspond to the genes annotated in the *I. scapularis* genome as “peptidase”, “protease” or “proteinase” that are different from the hemoglobinolytic enzymes. The three genes up-regulated and down-regulated with the highest fold change in response to *A. phagocytophilum* infection in tick midguts and salivary glands were selected on each protease category. Transcriptomics RNAseq data

from *A. phagocytophilum*-infected and uninfected tick samples were obtained from Ayllón et al. (2015). Only genes with statistically significant differences were included. Abbreviations: ID, gene (GenBank; http://www.ncbi.nlm.nih.gov) accession numbers; +, up-regulated genes in infected vs. uninfected ticks; -, down-regulated genes in infected vs. uninfected ticks; *Genes that appeared in both tick midguts and salivary glands.

Table S3. Percent homology for selected differentially represented host proteins.

Hosts[1]	Midguts						Salivary glands	
	HMG[2]	SA8	SA12	SERP	H2B	AP-1	TOPO1	IgHC
Ovis aries[3]	-	-	-	-	-	-	-	-
Homo sapiens	86.62	71.59	65.22	62.82	100	99.68	96.73	59.52
Equus caballus	88.03	84.27	77.17	70.51	100	99.58	97.07	64.19
Bos taurus	90.85	92.13	83.70	85.90	100	99.68	98.17	85.30
Canis lupus familiaris	80.99	80.90	67.39	70.36	100	99.79	97.00	58.78
Cervidae	82.98	NF[4]	NF	NF	NF	91.67	NF	40.85
Peromyscus	83.80	65.17	42.39	60.67	100	99.79	97.11	44.51
Microtus	82.39	61.80	42.39	60.41	100	99.47	95.95	NF
Tamias	82.39	NF	NF	32.61	NF	NF	NF	NF
Procyon	82.27	NF	NF	NF	NF	NF	NF	NF
Passeriformes	70.42	35.96	46.74	45.64	96.03	98.53	92.15	33.66

The most over-represented and under-represented sheep host proteins in *A. phagocytophilum*-infected *I. scapularis* midguts (fold change > 9, log2-fold >3.2 or <-3.2) and salivary glands (fold change > 6.5, log2-fold >2.7 or <-2.7) when compared to uninfected controls were selected for analysis.

[1]Hosts were selected based on their role as natural hosts for *A. phagocytophilum* and *I. scapularis* or for suffering granulocytic anaplasmosis after infection with *A. phagocytophilum* transmitted by *I. scapularis*. Hosts are represented at the order (Passeriformes/bird) family (Cervidae/deer), genus (Peromyscus/deer mice, Microtus/voles, Tamias/chipmunk and Procyon/raccoon) and species (*Ovis aries*/sheep, *Homo sapiens*/man, *Equus caballus*/horse, *Bos taurus*/caw and *Canis lupus familiaris*/dog) levels.

[2]Protein names were abbreviated as HMG (Hemoglobin subunit alpha; P21379), SA8 (S100 calcium-binding protein A8; W5NQK9), SA12 (S100 calcium–, zinc– and copper–binding protein A12; W5NQJ0), SERP (Serpin-like protein; W5P9M9), H2B (Histone H2B; P62808), AP-1 (Clathrin-associated adaptor protein complex 1 (AP-1) subunit beta-1; Q08DS7), TOPO1 (Topoisomerase I; F1MN93) and IgHC (Ig heavy chain; G5E5T5).

[3]Percent of protein identity was calculated using Clustal2.1 and taking *Ovis aries* homologs as reference.

[4]NF: No homolog was found using BLAST at http://www.ncbi.nlm.nih.gov.

Methods for the determination of protein homology for selected sequences. Major domestic and wild vertebrate hosts for *I. scapularis* that have been reported to be infected with *A. phagocytophilum* were obtained from previous publications (Keesing et al. 2012; Stuen et al. 2013; Dugat et al. 2015; Kocan et al. 2015; de la Fuente et al. 2015; Estrada-Peña et al. 2015). Four major domestic hosts were identified: *Ovis aries* (Oar), *Equus caballus* (Eca), *Canis lupus familiaris* (Clf) and *Bos taurus* (Bta) (Kocan et al. 2015). Wild hosts were identified belonging to the order Passeriformes (Keesing et al. 2012), the family Cervidae and the genera *Peromyscus*, *Microtus*, *Tamias* and *Procyon* (Stuen et al. 2013; Dugat et al. 2015). *Homo sapiens* (Hsa) was also included in the analysis as an accidental host for *I. scapularis* that can be infected by *A. phagocytophilum* causing HGA (Kocan et al.

2015). The most over-represented and under-represented sheep host proteins in *A. phagocytophilum*-infected *I. scapularis* midguts (fold change > 9, log2-fold >3.2 or <-3.2) and salivary glands (fold change > 6.5, log2-fold >2.7 or <-2.7 (see Dataset S1 in the supplemental material) when compared to uninfected controls were selected for analysis. Protein sequences from hemoglobin subunit alpha (HMG), S100 calcium-binding protein A8 (SA8), S100 calcium–, zinc– and copper–binding protein A12 (SA12), Serpin (SERP), histone H2B (H2B), clathrin-associated adaptor protein complex 1 (AP-1) subunit beta-1 (AP-1), topoisomerase I (TOPO1) and immunoglobulin heavy chain (IgHC) of *O. aries* were obtained from GenBank (http://www.ncbi.nlm.nih.gov). Homolog search of the above proteins was performed against the GenBank databases: *Equus caballus* (taxid: 9796), *Canis lupus familiaris* (taxid: 9615), *Bos taurus* (taxid: 9913), Passeriformes (taxid: 9126), Cervidae (taxid: 9850), *Peromyscus* (taxid: 10040), *Microtus* (taxid: 10053), *Tamias* (taxid: 13712) and *Procyon* (taxid: 9653) using Protein-Protein Basic Local Alignment Search Tool (BLASTP) from BLAST (Altschul et al. 1990; Madden et al. 1996) and *O. aries* sequences as "queries". The sequences with the lowest E–value were selected for further analysis. Accession numbers of protein sequences identified using the above procedure were HMG (Oar, CAA49750; Eca, CAA30097; Clf, NP_001257814; Bta, CAB56829; Cervidae, P01972; *Peromyscus*, ABN71080; *Microtus*, XP_005349096; *Tamias*, AGU68546; *Procyon*, P18977; Passeriformes, ALG03085 and Hsa, ABF56145), SA8 (Oar, XP_004002572; Eca, XP_001493639; Clf, NP_001139616; Bta, NP_001107197; *Peromyscus*, XP_006976290; *Microtus*, XP_005367318; Passeriformes, XP_008926848 and Hsa, NP_001306126), SA12 (Oar, XP_004002571; Eca, XP_001494448; Clf, XP_003434953; Bta, ACJ06402; *Peromyscus*, XP_006992615; *Microtus*, XP_005360276; Passeriformes, XP_014129499 and Hsa, NP_005612), SERP (Oar, XP_004020624; Eca, XP_014583359; Clf, XP_541074; Bta, NP_001193642; *Peromyscus*, XP_006993755; *Microtus*, XP_005368788; *Tamias*, BAD06478; Passeriformes, XP_005481730 and Hsa, AAQ04218), H2B (Oar, XP_004019078; Eca, XP_001497452; Clf, XP_545389; Bta, NP_001032546; *Peromyscus*, XP_006988912; *Microtus*, XP_005354996; Passeriformes, XP_002192965 and Hsa, NP_003509), AP-1 (Oar, XP_004012503; Eca, XP_001503974; Clf, XP_853723; Bta, NP_001068593; Cervidae, AAD41905; *Peromyscus*, XP_006995370; *Microtus*, XP_005349419; Passeriformes, XP_005495081 and Hsa, BAF82217), TOPOI (Oar, XP_011974341; Eca, XP_005604687; Clf, XP_534420; Bta, NP_001193416; *Peromyscus*, XP_006971113; *Microtus*, XP_005363050; Passeriformes, XP_005490040 and Has, NP_003277) and IgHC (Oar, S25705; Eca, AAU09792; Clf, P01874; Bta, AAN60017; Cervidae, ABO16483; *Peromyscus*, AAR21580; Passeriformes, XP_014117986 and Has, AAS01770). Percent identity matrices were generated for each group of homologs using Clustal 2.1 implemented in Clustal Omega (Sievers et al. 2011). Only pairwise comparisons including *O. aries* sequences were considered (Table 1).

References

Altschul SF, Gish W, Miller W, Myers EW, Lipman DJ. 1990. Basic local alignment search tool. J Mol Biol 215:403-410.

de la Fuente J, Estrada-Peña A, Cabezas-Cruz A, Brey R. 2015. Flying ticks: anciently evolved associations that constitute a risk of infectious disease spread. Parasit Vectors 8:538.

Dugat T, Lagrée AC, Maillard R, Boulouis HJ, Haddad N. 2015. Opening the black box of *Anaplasma phagocytophilum* diversity: current situation and future perspectives. Front Cell Infect Microbiol 5:61.
Estrada-Peña A, de la Fuente J, Ostfeld RS, Cabezas-Cruz A. 2015. Interactions between tick and transmitted pathogens evolved to minimise competition through nested and coherent networks. Sci Rep 5:10361.
Keesing F, Hersh MH, Tibbetts M, McHenry DJ, Duerr S, Brunner J, Killilea M, LoGiudice K, Schmidt KA, Ostfeld RS. 2012. Reservoir competence of vertebrate hosts for *Anaplasma phagocytophilum*. Emerg Infect Dis 18:2013-2016.
Kocan KM, de la Fuente J, Cabezas-Cruz A. 2015. The genus *Anaplasma*: new challenges after reclassification. Rev Sci Tech 34:577-786.
Madden TL, Tatusov RL, Zhang J. 1996. Applications of network BLAST server. Methods Enzymol 266:131-141.
Sievers F, Wilm A, Dineen D, Gibson TJ, Karplus K, Li W, Lopez R, McWilliam H, Remmert M, Söding J, Thompson JD, Higgins DG. 2011. Fast, scalable generation of high-quality protein multiple sequence alignments using Clustal Omega. Mol Syst Biol 7:539.
Stuen S, Granquist EG, Silaghi C. 2013. *Anaplasma phagocytophilum*--a widespread multi-host pathogen with highly adaptive strategies. Front Cell Infect Microbiol 3:31.
